# Author Correction: Temporal dynamics of uncertainty and prediction error in musical improvisation across different periods

**DOI:** 10.1038/s41598-025-88641-w

**Published:** 2025-02-21

**Authors:** Tatsuya Daikoku

**Affiliations:** 1 Graduate School of Information Science and Technology, The University of Tokyo, 7-3-1 Hongo, Bunkyo-Ku, Tokyo, 113-8656 Japan; 2 Centre for Neuroscience in Education, University of Cambridge, Cambridge, UK; 3 Center for Brain, Mind and KANSEI Sciences Research, Hiroshima University, Hiroshima, Japan

Correction to: *Scientific Reports* 10.1038/s41598-024-73689-x, published online 27 September 2024

The original version of this Article contained errors. Due to a partial corruption in the dataset file, the recording year of some of the songs included was incorrect.

As a result, in the Abstract,

“This study employed the Hierarchical Bayesian Statistical Learning (HBSL) model to analyze a corpus of 456 Jazz improvisations, spanning 1905 to 2009, from 78 distinct Jazz musicians.”

now reads:

“This study employed the Hierarchical Bayesian Statistical Learning (HBSL) model to analyze a corpus of 456 Jazz improvisations, spanning 1925 to 2009, from 78 distinct Jazz musicians.”

In the Introduction, under the subheading ‘Purpose of the present study’,

“Hierarchical Bayesian Statistical Learning Model) of tone sequence from a corpus of 456 Jazz improvisation played from 1905 to 2009 years by 78 different Jazz musicians, as the training data.”

now reads:

“Hierarchical Bayesian Statistical Learning Model) of tone sequence from a corpus of 456 Jazz improvisation played from 1925 to 2009 years by 78 different Jazz musicians, as the training data.”

In the Methods section, under the subheading ‘Materials and procedure’,

“The HBSL model computes the Shannon information content and entropy based on transitional probabilities^32^ of tone sequence from a corpus of 456 Jazz improvisation (The Jazzomat Research Project, https://jazzomat.hfm-weimar.de/index.html) played from 1905 to 2009 years by 78 different Jazz musicians, as the training data.”

now reads:

“The HBSL model computes the Shannon information content and entropy based on transitional probabilities^32^ of tone sequence from a corpus of 456 Jazz improvisation (The Jazzomat Research Project, https://jazzomat.hfm-weimar.de/index.html) played from 1925 to 2009 years by 78 different Jazz musicians, as the training data.”

In addition, the year labels in Figs. 2, 3, 4 and 5, as well as the year range in the legends of Fig. 2 and Fig. 3 were incorrect.

The original Figs. 2, 3, 4 and 5 and accompanying legends appear below.

Finally, in the Supplementary Material 1 file the ‘Year’ graphs in the Supplementary Figure S2A, S2B and S3 were incorrect and have been removed.

The original Supplementary Material 1 file is provided below.

The original Article and accompanying Electronic supplementary material file have been corrected.

**Fig. 2:**
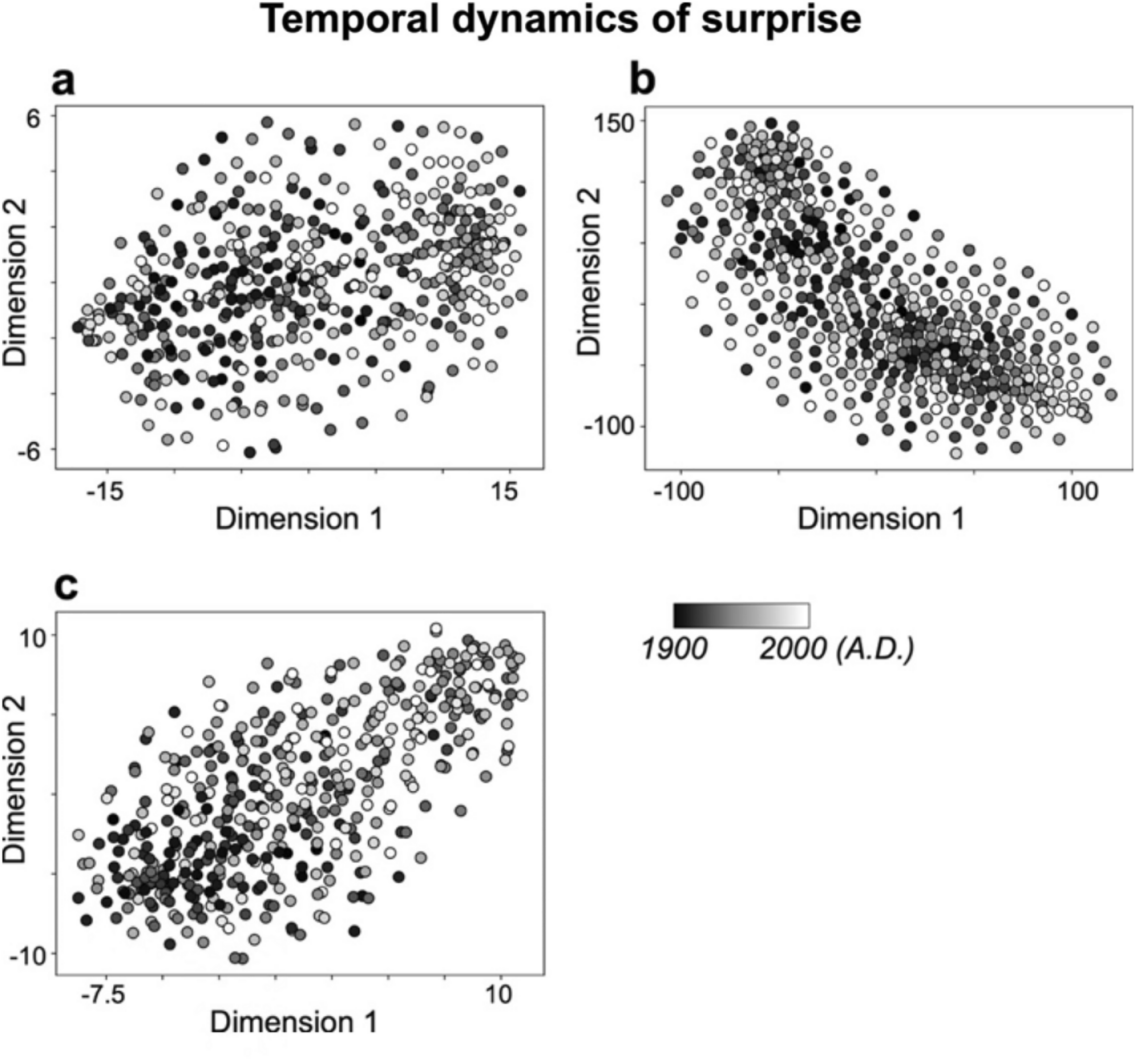
Characteristics of temporal dynamics of surprise (inverse of probability values) in pitch (**a**), rhythm (**b**), and pitch-rhythm (**c**) sequences, using tSNE. The dots that are close together indicate similar temporal dynamics of surprise in sequence of improvisation, while dots that are far apart indicate dissimilar temporal dynamics of surprise. Each dot represents a song from the years 1900 to 2000. The darker the color, the older the song (closer to 1900), and the lighter the color, the more recent the song (closer to 2000).

**Fig. 3:**
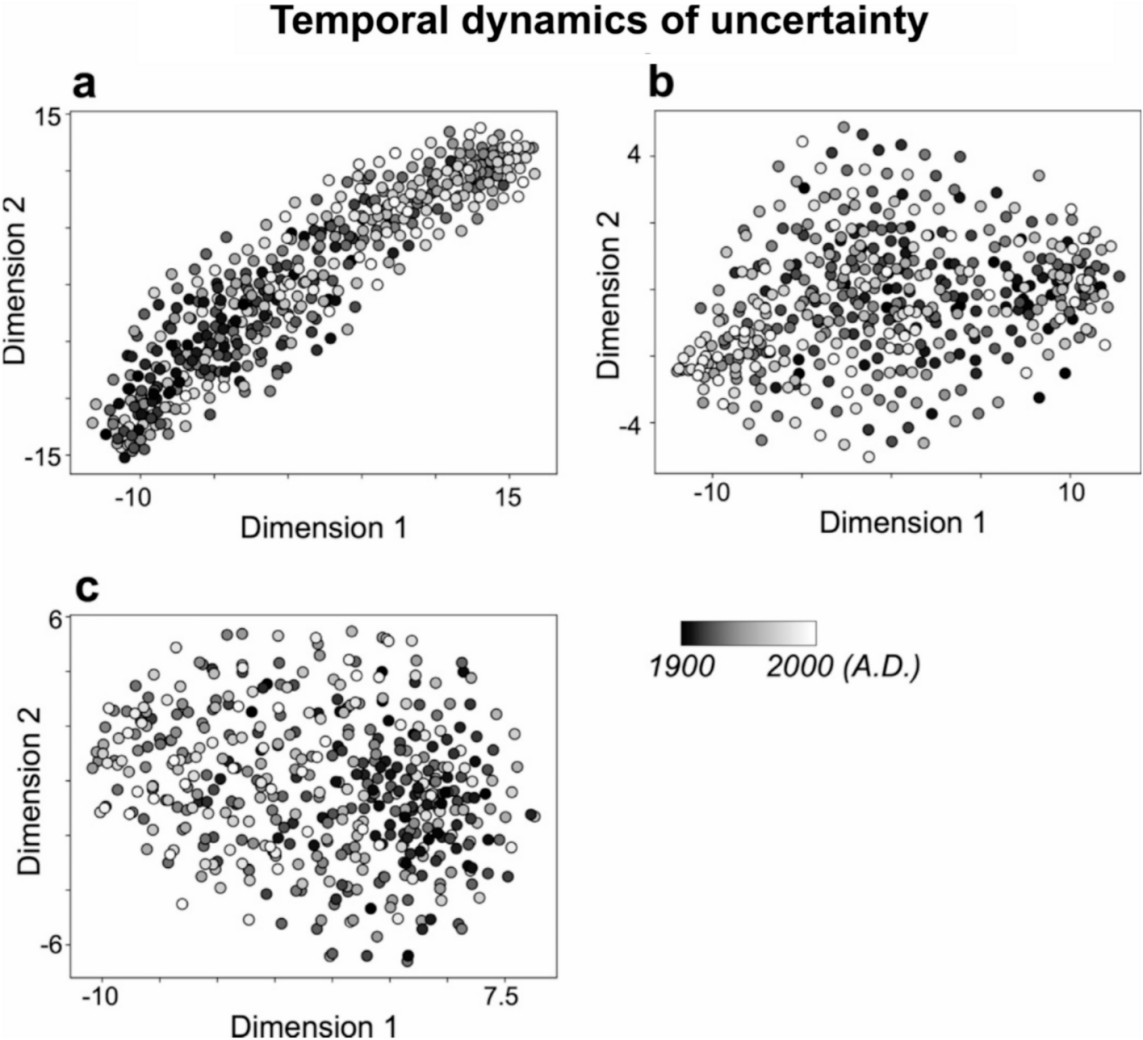
Characteristics of temporal dynamics of uncertainty (entropy values) in pitch (**a**), rhythm (**b**), and pitch-rhythm (**c**) sequences, using tSNE. The dots that are close together indicate similar temporal dynamics of uncertainty in sequence of improvisation, while dots that are far apart indicate dissimilar temporal dynamics of uncertainty. Each dot represents a song from the years 1900 to 2000. The darker the color, the older the song (closer to 1900), and the lighter the color, the more recent the song (closer to 2000).

**Fig. 4:**
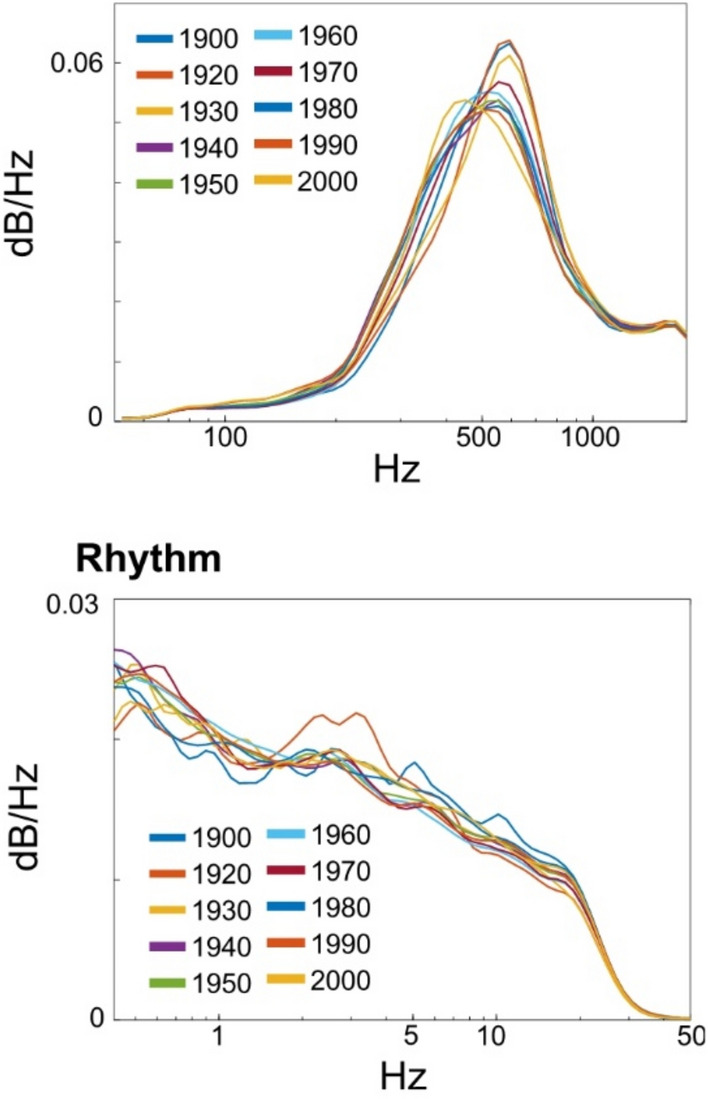
Acoustic properties of spectral frequency (pitch) and temporal frequency (rhythm, envelope of waveform) in each decade. The x and y axes represent frequency and dB/Hz, respectively. Regardless of music, they showed similar frequency distribution in both pitch and rhythm dimensions.

**Fig. 5:**
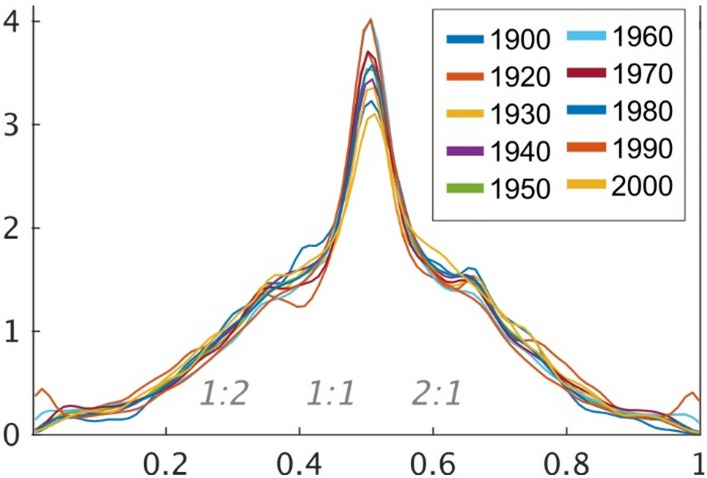
Probabilistic density for cycle rate in the modulation envelope (rhythm) in each decade. The Y-Axes show the Probabilistic density. The X-Axes show the rhythm rate. Based on the formula of c1/(c1 + c2), the 0.5 (1:1) rhythm rate indicates that the relationship between the length of a given cycle (AM c1) and that of the subsequent cycle (AM c2) is equivalent, while a 1:2 rate refers to a situation where the length of the succeeding cycle is twice that of the preceding cycle. The probability densities of a 1:1 Rate were stronger than the other rates. Then, the probability densities of the 1:2 and 2:1 rate was also relatively stronger than the other rates.

## Supplementary Information

Supplementary Information.

