# Supplementary material for: Temporal dynamics of uncertainty and prediction error in musical improvisation across different periods
Source: Sci Rep. 2024 Sep 27;14:22297. doi: 10.1038/s41598-024-73689-x (PMC11437158; doi:10.1038/s41598-024-73689-x)

**Figure. S1: Characteristics of temporal dynamics of surprise (inverse of probability values) in pitch (a), rhythm (b), and pitch-rhythm (c) sequences, using tSNE.**

**Figure S2A. Acoustic properties of temporal frequency (rhythm, envelope of waveform) in each decade.**

**
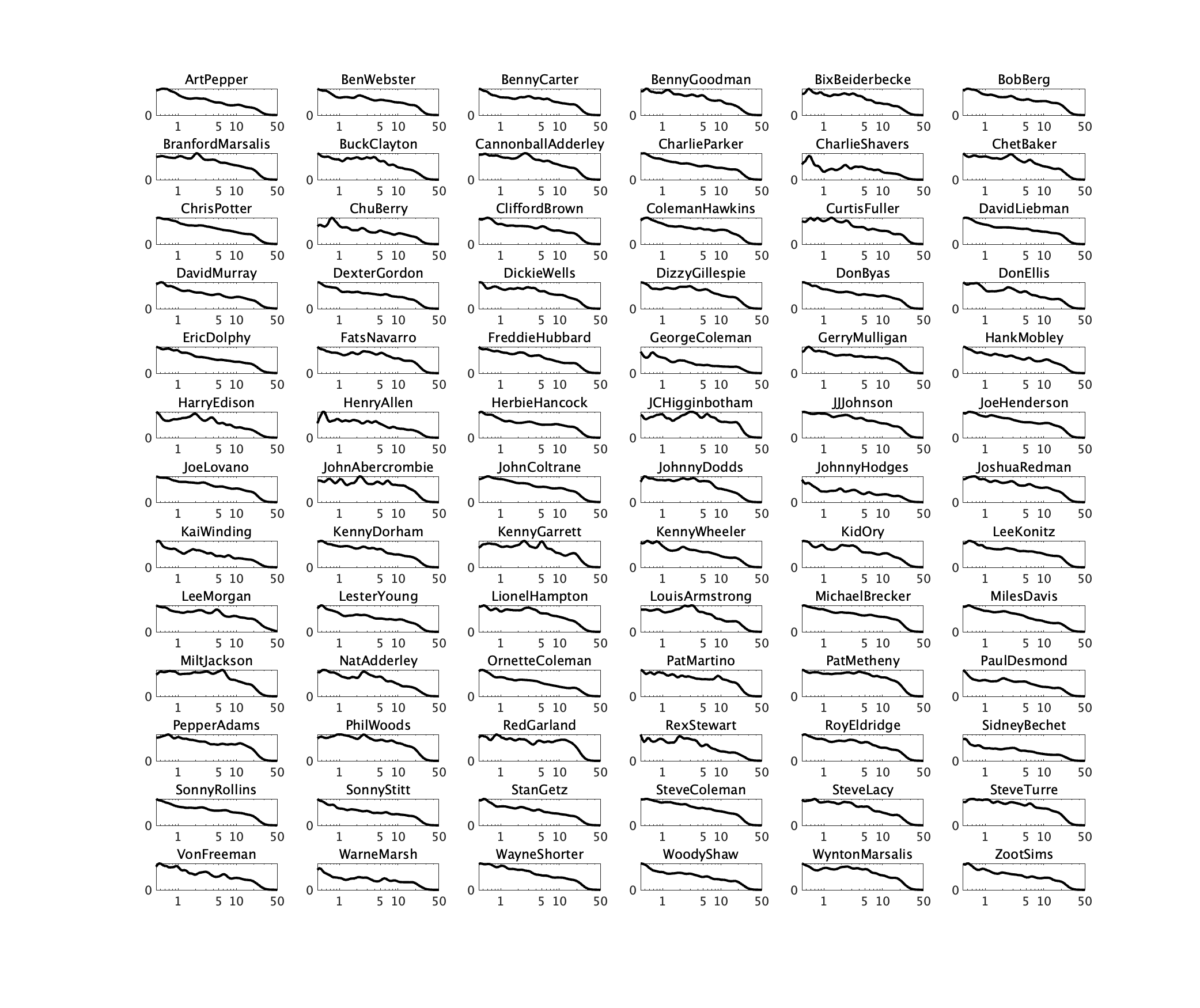
Player**

**Style**


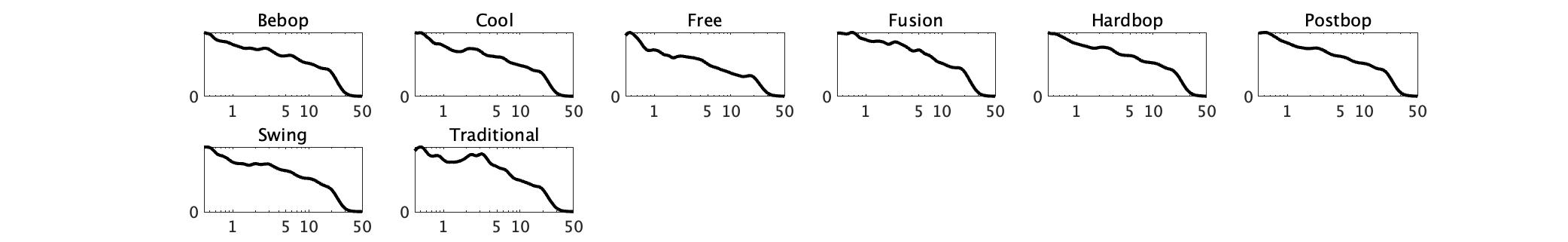


**Instrument**


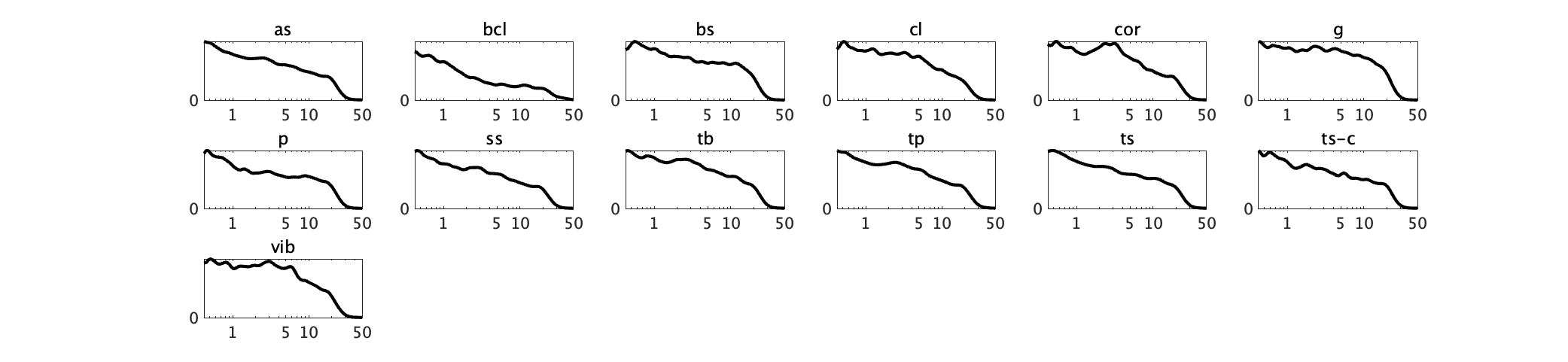


**Figure S2B. Acoustic properties of spectral frequency (pitch) in each decade.**

**Player
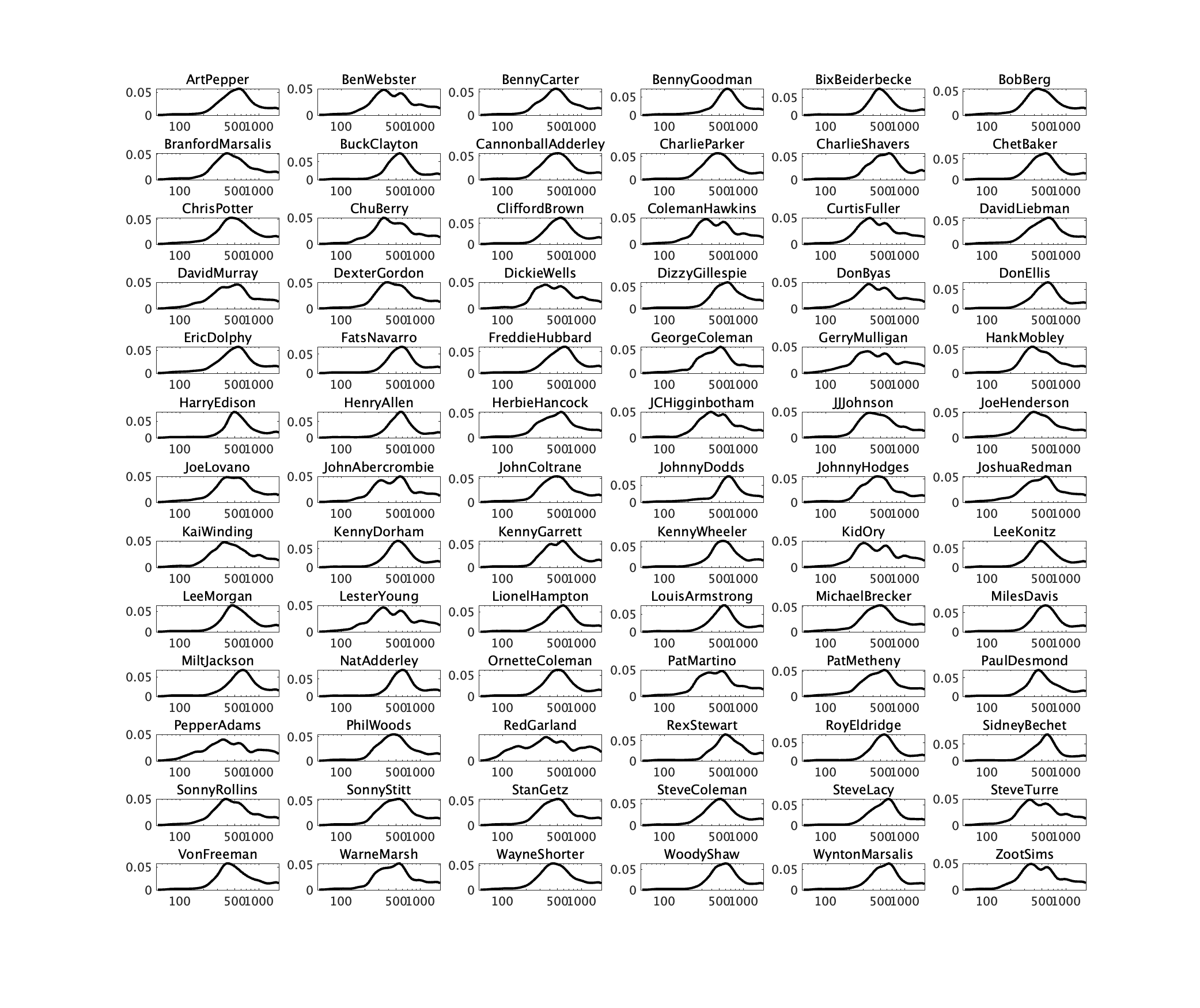
**

**Style**


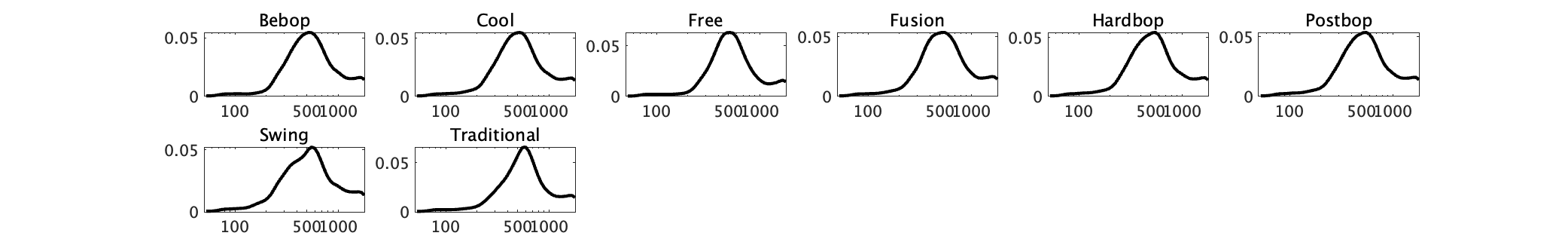


**Instrument**


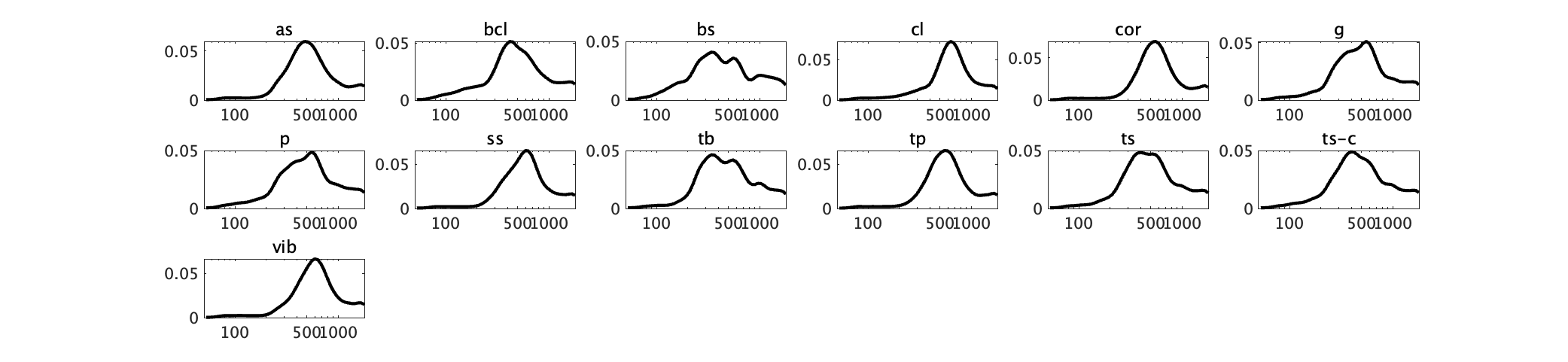


**Figure. S3: Probabilistic density for cycle rate in the modulation envelope (rhythm) in each decade.**


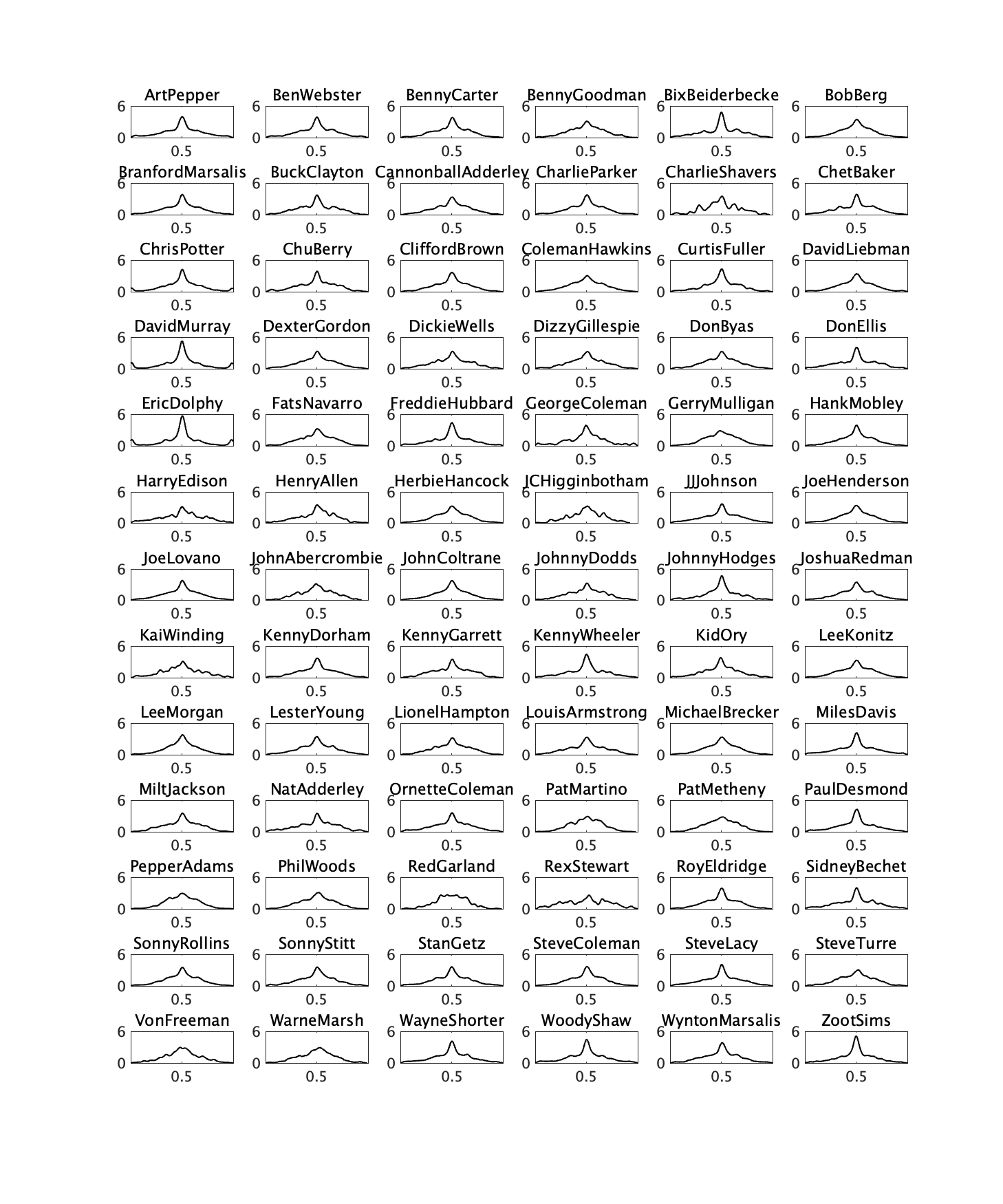
**Player**

**Style**


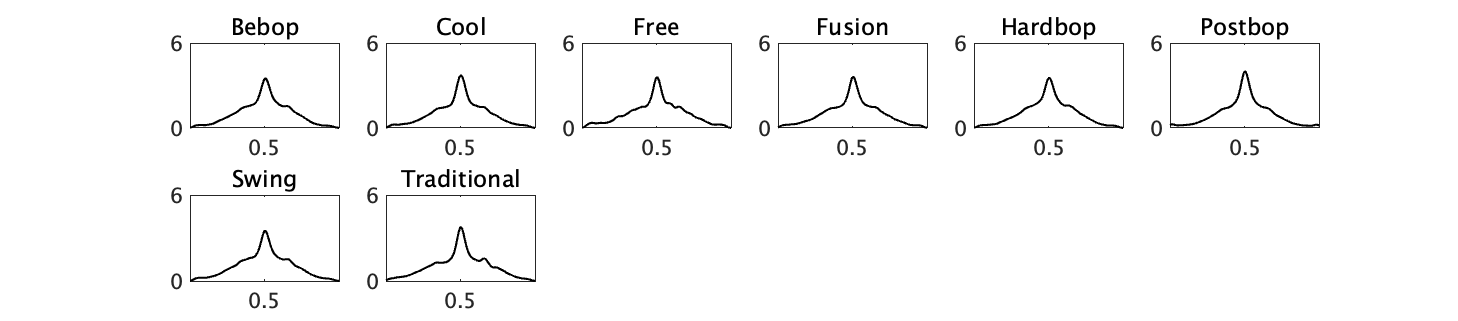


**Instrument**


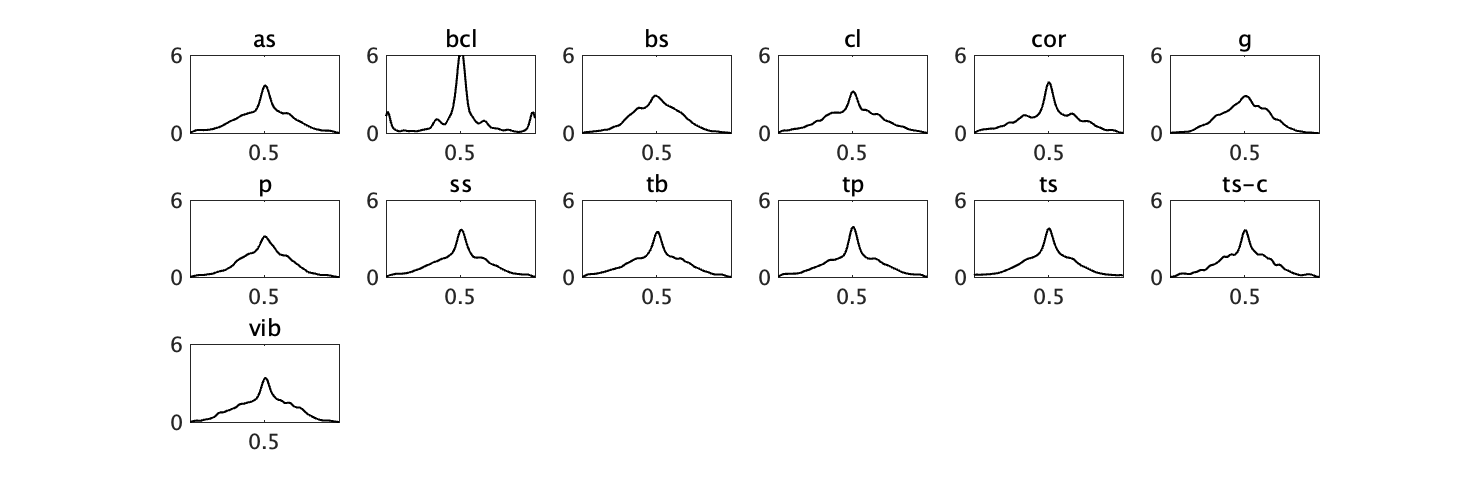

Supplement: Supplementary file 1 — Supplementary Material 1 [file 41598_2024_73689_MOESM1_ESM.docx]
